# Three topological features of regulatory networks control life-essential and specialized subsystems

**DOI:** 10.1038/s41598-021-03625-w

**Published:** 2021-12-20

**Authors:** Ivan Rodrigo Wolf, Rafael Plana Simões, Guilherme Targino Valente

**Affiliations:** 1 Department of Bioprocess and Biotechnology, School of Agriculture, São Paulo State University (UNESP), Botucatu, São Paulo 18610-034 Brazil; 2 Medical School, Sao Paulo State University (UNESP), Botucatu, São Paulo 18618-687 Brazil; 3 Max-Planck-Institut für Herz- und Lungenforschung, Max Planck Institute, 61231 Bad Nauheim, Hessen Germany

**Keywords:** Evolutionary biology, Gene regulation, Computational models, Data mining, Gene regulatory networks, Machine learning, Network topology

## Abstract

Gene regulatory networks (GRNs) play key roles in development, phenotype plasticity, and evolution. Although graph theory has been used to explore GRNs, associations amongst topological features, transcription factors (TFs), and systems essentiality are poorly understood. Here we sought the relationship amongst the main GRN topological features that influence the control of essential and specific subsystems. We found that the K_nn_, page rank, and degree are the most relevant GRN features: the ones are conserved along the evolution and are also relevant in pluripotent cells. Interestingly, life-essential subsystems are governed mainly by TFs with intermediary K_nn_ and high page rank or degree, whereas specialized subsystems are mainly regulated by TFs with low K_nn_. Hence, we suggest that the high probability of TFs be toured by a random signal, and the high probability of the signal propagation to target genes ensures the life-essential subsystems’ robustness. Gene/genome duplication is the main evolutionary process to rise K_nn_ as the most relevant feature. Herein, we shed light on unexplored topological GRN features to assess how they are related to subsystems and how the duplications shaped the regulatory systems along the evolution. The classification model generated can be found here: https://github.com/ivanrwolf/NoC/.

## Introduction

Living cells are machines ruled by miscellaneous interactions among their components. The protein–protein, metabolic, signaling, regulatory, and other biological networks can be modeled as graphs^1^ organized in modules (subsystems or sub-networks)^2^. An in-deep knowledge concerning the organization of these networks would lead to a better comprehension of DNA repair mechanisms^3^, cellular differentiation^4^, metabolism^5^, evolution^6,7^, and could drive technological advances in many fields^1,8–10^.

Genetic regulatory networks (GRNs) represent target gene regulations mediated by transcription factors (TFs)^9,11^. TFs are elements responsible for activating or repressing the target gene expression by physical interaction onto genomic binding sites (regulatory elements) or binding to regulatory proteins^12^. GRNs interconnect subsystems to control cell physiology and environmental response^13–15^. Therefore, GRNs play essential roles in development^16^, phenotypic plasticity^7,17^, disease^11^, and evolution^18,19^. Mutations in regulatory regions may impact GRN evolution^13,20,21^. Modification in regulatory elements can lead to variations in phenotypes^22^, and mutations can generate cryptic TF binding sites^21^. The TFs recognize degenerated DNA motifs surrounding genes leading to TFs overlapping onto the same genomic regions^23^. This overlap may start the pervasive transcription (the transcription of different RNAs from the same site)^21^, which may result in morphological evolution^22^. Additionally, genome and gene duplications are important factors for the GRN evolution^14,16,20,24–26^ since it leads to TF duplication and bifunctionality^24,25,27^. For instance, after duplications, maintenance of ancient interactions correspond to the evolution of ~ 90% of regulatory interactions in *E. coli* and *S. cerevisiae*^25^. Then, genomic changes can lead to network rewiring^28,29^ and network topological features changing.

TFs and target genes in GRNs are modeled in graphs as vertices (or nodes) and their interactions as edges (or links). Network centralities can be used to weigh the significance of a node^30–33^. For instance, housekeeping genes have higher centralities than other genes^33^, and disease-related genes have specific ranges of cluster coefficient and betweenness centrality^34,35^.

Although plenty of discussions about GRN is available, relationships amongst topological features, TFs, and subsystems essentiality are still murky. Moreover, how the significance of topological features may change along the GRN evolution is unclear. Herein, the goals were to assess the most relevant topological features of regulators (e.g., TFs) and target genes from GRNs, to understand how these features evolve, and their relationship to essential or specialized subsystems. We found that K_nn_ (the average nearest neighbor degree), page rank, and degree solely split regulators from targets. Simulations showed that duplicating the targets decreases the regulator’s K_nn_, whereas duplicating the regulators increases the regulator’s K_nn_. Furthermore, we showed that TF-hubs with low K_nn_ (such as the ones that had duplicated targets) work on specialized subsystems, whereas TFs with intermediate K_nn_ and high page rank or degree control the life-essential subsystems; these features (mainly the high page rank) assure the essential subsystems robustness against random perturbation. Finally, we found that the GRN features mentioned are conserved and primary traits in cell development.

## Results

We used GRNs of *Escherichia coli*, *Saccharomyces cerevisiae*, *Drosophila melanogaster*, *Arabidopsis thaliana*, *Homo sapiens* and mESC cells (the mESC set was used only as a test set) to seek the main GRN topological features and how the ones are related to each other. After the filtering steps, 49,801 regulatory interactions were selected from species-specific sets, with a total of 12,319 nodes (instances) (1073 regulators and 11,246 targets) (Table 1, Supplementary Table S1). The data composed 12 balanced training sets, 11 out of them had 1938 instances, and only 1 had 966 instances (Supplementary Data S1). The number of genes in each network represented up to 51.17% of all genes in each genome (Table 1). The scale-free property usually does not emerge in sub-nets and smaller networks^36,37^. However, each filtered network fits a power-law function (R^2^ ≈ 1) (Supplementary Fig. S1), evidencing they are scale-free since the power-law maintains the same functional form at all scales. Therefore, the filtered networks present the main topological properties even though not harboring all genes. Overall, the scale-free property is a relevant feature of biological networks, including GRNs, providing network resilience against random node removal and fitting the data of genome evolution by gene duplication^1,17,24,38–47^.

**Table 1:** The number of interactions, regulators, and targets of analyzed GRNs.

Organism/cell type	Raw interaction	Interaction*	Target*	Regulator*	Total of instances*	References	Num. genes	Num. TFs; Reference	% genes used
*E. coli*	4490	3744	1594	197	1791	^75^	4464	207^76^	40.12
*S. cerevisiae*	17,030	17,030	3150	149	3299	^77^	6446	301^17^	51.17
*D. melanogaster*	19,657	14,319	767	114	881	^78^	17,532	1052^17^	5.02
*A. thaliana*	18,772	5117	3428	307	3735	^79^	33,467	2451^17^	11.16
*H. sapiens*	106,096	9591	2307	306	2613	^78^	42,220	1639^80^	6.18
mESC**	110,517	110,517	21,025	40	21,065	^81^	–	–	–
mESC-J1**	17,422	17,422	8148	6	8154	^81^	–	–	–
mESC-V6.5**	5675	5675	2758	3	2761	^81^	–	–	–
mESC-E14**	361	361	361	1	362	^81^	–	–	–

*Number of interactions and nodes after filtering.

**Datasets exclusively used as test sets. The number of genes per species were retrieved from NCBI (accession numbers GCF_000005845.2, GCF_000146045.2, GCF_000001215.4, GCF_000001735.4, and GCF_000001405.39). The “Num. TFs” depicts the number of transcription factors of each species. The “% genes used” are the proportion between the “Total of instances” and the “Num. of genes”.

**Figure 1:**
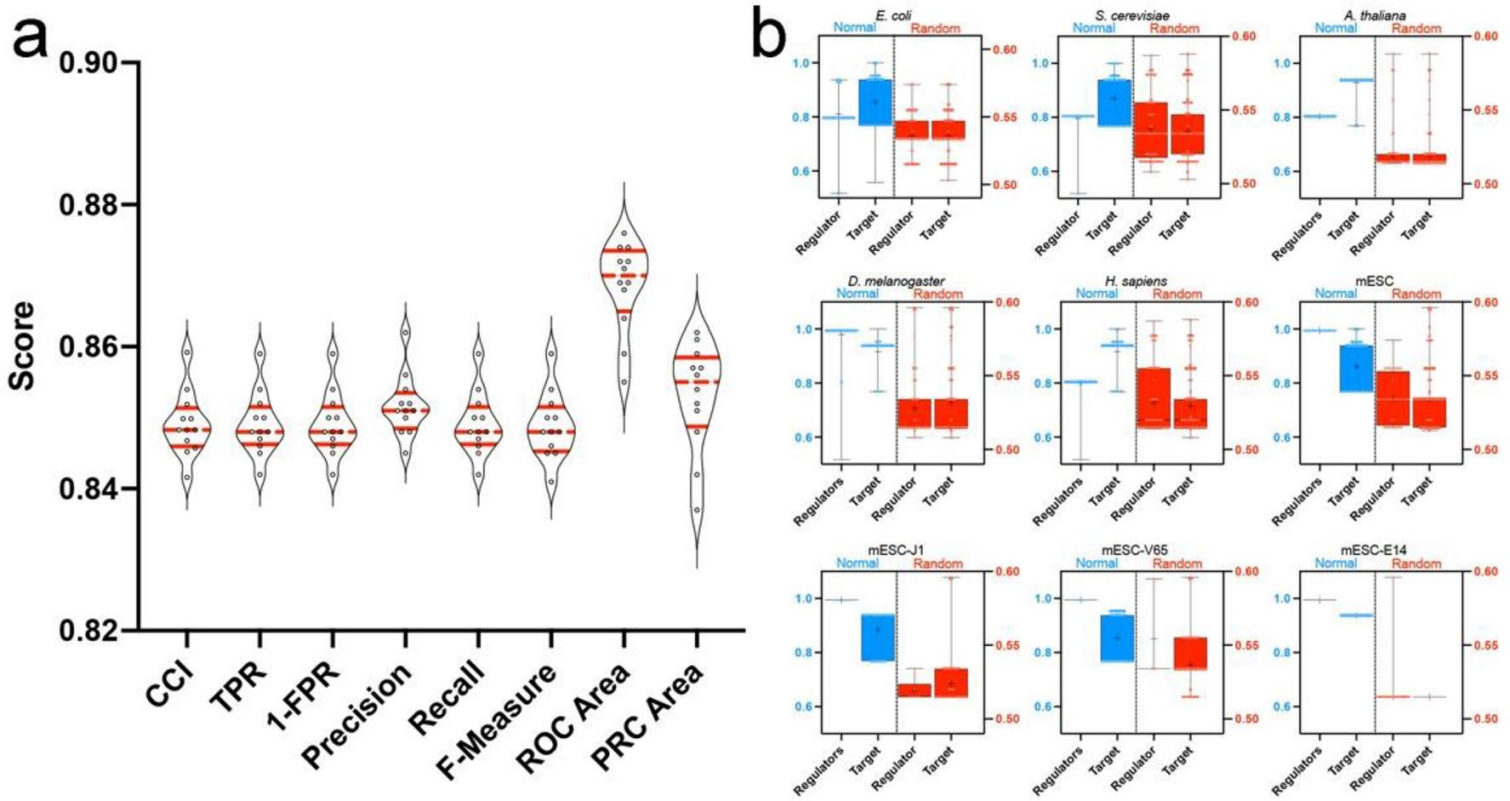
Predictive performances. (**a**) Predictive performances during supervised learning. *CCI* correctly classified instances, *TPR* true positive rate, *1-FPR* one minus false positive rate, *MCC* Matthews correlation coefficient, *ROC area* receiver operating characteristic area under the curve, *PRC area* precision-recall curve; (**b**) predictive score of the consensus models over each test set. Blue boxes and the Y-left axis depict the classification using the normal consensus model (only the scores from CCI were plotted), whereas the red boxes and Y-right axis depict classification using the random model. The “+” indicates the mean. The Mann–Whitney test showed a p-value < 0.001 for all comparisons.

The K_nn_ (the average nearest neighbor degree), page rank, and degree ranked as the most important attributes (the most relevant node’s topological features) during the attribute selection step (Supplementary Table S2): the ones were used to build the machine learning models. Decision trees ranging from 9 to 15 leaves (Supplementary Data S1, Supplementary Fig. S2) were obtained based on the 3 attributes mentioned, scoring an average of correctly classified instances (CCI) of 84.91% and a ROC average of 86.86% (Fig. 1a). A total of 44,661 instances composed the whole test set. The independent classification of each test set by the normal consensus model provided a CCI ranging from 68.23% to 100%, with high predictive scores for all cases (≥ 0.8). Training and classifying randomized sets provided low predictive performances: the training had an average of CCI = 51.82% and ROC of 51%, the test set classification score reached ~ 0.5 (Fig. 1b), and more complex trees (up to 17 leaves) were generated (Fig. 1b, Supplementary Table S3). The lower performance using the random data supports the reliability of the normal model.

**Figure 2:**
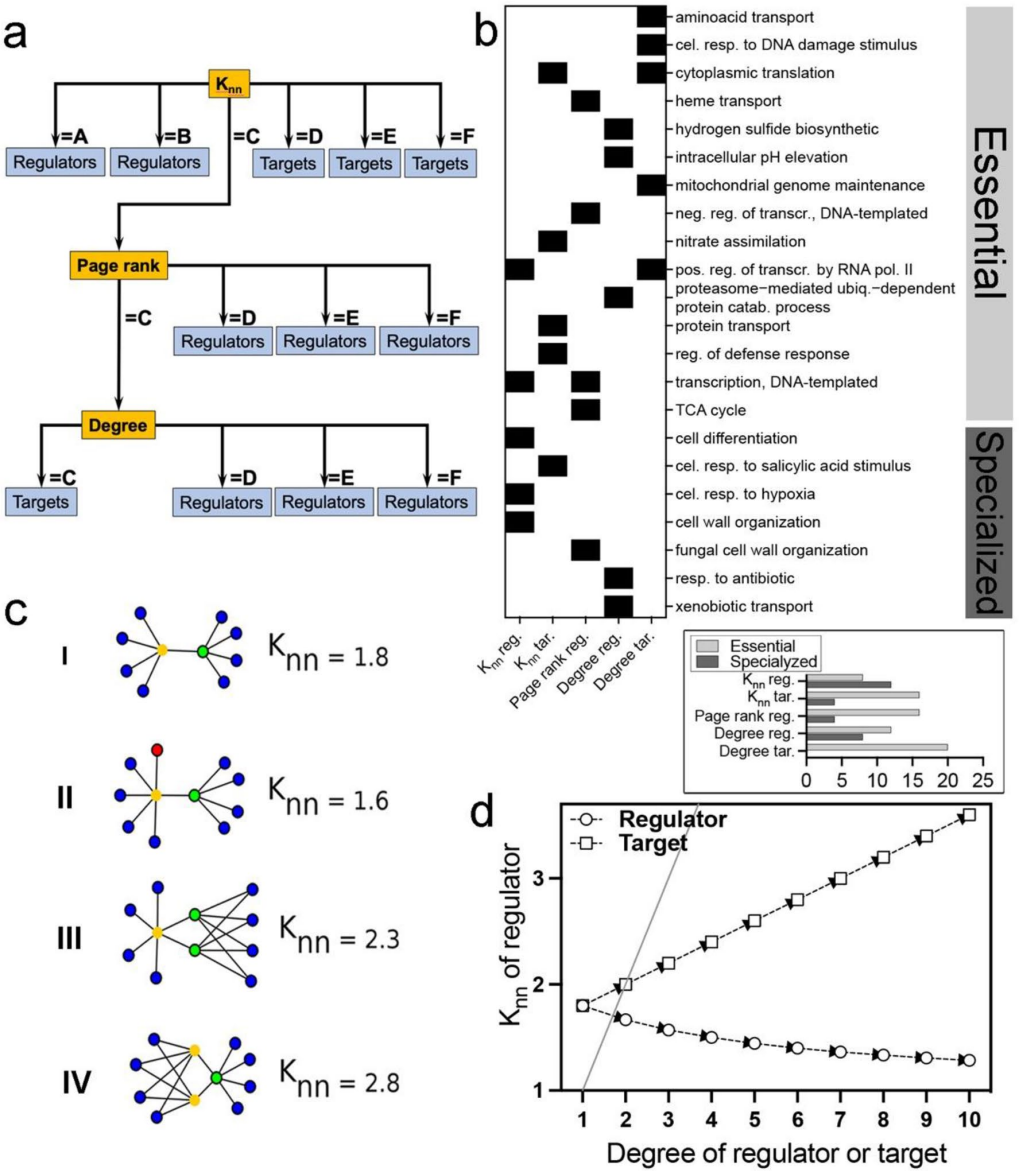
Decision tree, GO, and network simulation analysis. (**a**) The consensus tree which “A”, “B”, “C”, “D”, “E”, and “F” are the bins from the discretization step. Orange squares are the node’s features, and blue squares are the classified leaves; (**b**) the biological process (rows) of genes in tree’s leaves in (**a**) and the feature that leads to the leaves (K_nn_, degree, or page rank) (columns). The “reg.” means regulators, “tar.” means targets. The black box indicates the presence of a given GO term in genes at that tree leaves. The histogram in the box below the heatmap depicts the percentage of GO terms from genes that lie in each leaf type; (**c**) representation of hypothetical networks. The K_nn_ was calculated for the regulators (yellow nodes). Blue nodes are genes with just one connection. The red node depicts a blue node duplication. The green nodes represent other regulators or genes regulated by many regulators. “I”, “II”, “III”, and “IV” represents networks in an initial state, after a gene duplication or during pervasive transcription, after duplication of a different regulator, and after duplication of the regulator in which K_nn_ is calculated, respectively; (**d**) simulation of K_nn_ evolution of regulators from (**c**). The X-axis is the degree of targets and regulators, and the Y-axis is the regulator’s K_nn_. The diagonal grey line is the identity line (a line where every point has proximal X and Y coordinates), which by crossing only the second point, indicates divergencies since the beginning of the simulation.

The small (“A” and “B”) and high (“D-F”) K_nn_ are related to regulators and targets, respectively. A confusion area (K_nn_ depicted as “C”) leads the model to use the page rank to classify the other instances. Then, nodes with high page rank “D–F” are classified as regulators, whereas the small value (depicted as “C”) is a confusion area solved by the degree. Finally, small (“C”) and high (“D–F”) degrees are used as rules to classify targets and regulators, respectively (Fig. 2a, Supplementary Data S1).

The classified genes that lie in target and regulator leaves of the consensus tree (Fig. 2a) are related to cellular processes such as transcription, protein transport, energy metabolism, cell differentiation, cell wall organization, among others. We highlight that specialized processes (e.g., cell differentiation) are mainly related to regulators with low K_nn_, whereas essential processes are mainly related to regulators with high page rank or degree (Fig. 2a,b, Supplementary Fig. S3).

Network dynamic simulation was used to assess how the K_nn_ emerges as an important feature of GRNs’ nodes. Simulating the evolution of a hypothetical initial network (Fig. 2c I) under the hypothesis of pervasive transcription and target duplication of a given regulator (Fig. 2c II–IV), we found that increasing the degree of regulators (the duplication of targets) (Fig. 2c II) lead to a smooth decreasing of regulator’s K_nn_ (Fig. 2d). Conversely, increasing the degree of targets (for instance, by duplicating the regulators) (Fig. 2c III,IV) increases the regulator’s K_nn_ (Fig. 2d), indicating duplication as an important factor influencing the K_nn_.

## Discussion

Here, the decision tree showed the relationship among the essential topological features of regulators and targets in GRNs, allowing us to discuss how GRNs are structured and presenting biological insights concerning these topologies. Overall, K_nn_, page rank, and degree solely distinguish regulators from targets. The relevance of these GRN features seems evolutionary conserved and may be a primary cell feature, although more species and experiments need to be evaluated to better support this conclusion. Many genes at the decision tree’s leaves fit essential functions observed in the minimum genome^48,49^, and we could assess how topologies are related to these subsystems. Simulations depicted how the K_nn_ emerges as the most significant feature reported by the decision trees.

Regulators usually are hubs (highly connected nodes) in GRNs^50^. Our simulation evidenced that increasing the degree of a regulator reduces its K_nn_. Thereby TF-hubs have small K_nn_ meaning their targets have low connections. K_nn_ of a node is the average degree of its neighbors^39^, and the presence of reduced K_nn_ and degree suggest that high degree nodes may be binding to low degree nodes^51^. Interestingly, our tree did not depict any regulator with high K_nn_. Altogether, we suggest that TF-hubs (such as those with duplicated targets) work early on regulatory cascades and probably control specialized modules, which have fewer connections. Indeed, most of TFs with low K_nn_ seems to regulate specialized subsystems, and only two gene ontology (GO) terms of this kind of regulators (low K_nn_) are essential subsystems (“pos. reg. of transcr. by RNA pol. II”, and “transcription, DNA-templated”) (see “K_nn_ reg.” in Fig. 2b, Supplementary Fig. S3). Remarkably, the targets with high K_nn_ (the ones bind to high degree nodes) usually work on essential subsystems (see “K_nn_ tar.” in Fig. 2b). Hence, we suggest that a high K_nn_ for these targets may provide robustness against random perturbation, ensuring the indispensable reception of signals for these life-essential subsystems, such as expected for scale-free networks.

Our data evidenced that targets and regulators with intermediate K_nn_ values probably are connected to subsystems with similar topologies. Although the K_nn_ can not distinguish these nodes, the high page rank is a signature of these regulators. Interestingly, the regulators with high page rank usually control essential processes (e.g., transcription and TCA cycle) (see “Page rank reg.” in Fig. 2b, Supplementary Fig. S3). The page rank of a node is proportional to its importance, and a higher value indicates that more often signals randomly walking through the network will visit this node^31,39^. GRNs are closely linked to metabolic networks^52^. Thus, internal and external stimuli signals can efficiently reach regulators to trigger the transcription of genes related to the response mechanisms^53,54^. Therefore, we suggest that regulators of essential subsystems are prone to be activated by signals emitted from multiple network sources, assuring a faster signal response.

The targets with intermediary K_nn_ and the lowest page rank (depicted as “C” in the tree) have a low degree. The low degree is related to low page rank^55^. Therefore, we suggest that these targets (low degree) probably lie at the end of regulatory chains without massive links to allow the signal flow of regulatory information. Moreover, we suggest that the regulators with low page rank and high degree probably act, or connect, within densely connected subsystems (such as sub-circuits and gates^16^).

The good performance of the normal consensus model to classify the species-specific test sets indicates that the K_nn_, page rank, and degree are topological features conserved along the evolution. Notwithstanding, the good classification of GRNs from mouse embryonic stem cells also showed that these topological features arise as essential properties even before the cell differentiation, albeit a previous paper showed that the topological properties of TFs are different amongst tissues^56^, reflecting different cell states^34^.

Altogether, our model suggests that the high probability of TFs in a system be toured by a random signal (nodes with high page rank), and the high probability of signal propagation to target genes (nodes with high K_nn_) ensures robustness to the life-essential subsystems against random perturbation.

Our simulations preserving old interactions after duplications (such as pointed in GRNs of *E. coli* and *S. cerevisiae*^25^) showed that duplication is the main evolutionary process to prompt K_nn_ as the most important GRN feature, corroborating the relevance of duplications for GRN evolution. Redundancies allow for the evolution of regulators^57^ by diversifying signal or co-factor recognitions, by gain/loss of binding sites^27^, or by inducing pervasive transcription^21^. Furthermore, the duplication of regulators can lead to several combinations of expression regulation intensities^58^. Thus, new gene expression profiles may arise, avoiding the negative effects of regulatory changes^27,59^. Therefore, we suggest that duplicating the regulators and targets creates redundancies within GRNs, increasing the system robustness from random perturbations even though sometimes noticing a smooth shrinking of regulator’s K_nn_; this conclusion is also supported by classical findings of small-world effect and the networks growth model^60^.

After the duplication events, epigenetic changes may selectively silence duplicated genes^61^. Then, genomes go towards a reductive phase in which the adaptive genome streamlining or genetic material loss occurs^62^. Otherwise, K_nn_ would continuously grow, such as observed in our simulations. In plants, the differential expression of paralogs seems to influence gene retention after duplication^46^. Since the number of targets overcomes the number of regulators in our data, we hypothesized that the loss of regulators is more likely than targets. Finally, regulators kept until the final stages of genome reduction are probably conserved as an essential part of regulatory sub-circuits^13^; or the ones may be maintained by the neo-functionalization process^27,63^. The *Hox* gene cluster exemplifies the evolutionary events mentioned. This cluster harbors crucial transcription factors for body plan development in bilaterian animals^64^. Many species, such as *Danio rerio*, *Takifugu rubripes,* and *Mus musculus*, have multiple *Hox* clusters due to duplications. However, all clusters have undergone gene/cluster loss along the evolution^65,66^.

As far as we know, relationships between topological features of GRNs and subsystems and simulations depicting how duplications increase the importance of topological features were never assessed before: previous papers focus on mathematical properties of systems. Our data allowed us to suggest how specific systems emerged through evolution, the presence of some GRN’s features since the pluripotent state, and how gene duplication may be shaping different regulatory systems.

## Methods

### Parsing the regulatory networks and attributes calculation

The experimentally validated GRNs of *E. coli*, *S. cerevisiae*, *A. thaliana*, *D. melanogaster*, and humans were obtained from databases (Table 1); the ones are hereafter referred to as species-specific GRNs. The gene names of *E. coli* and *S. cerevisiae* were converted to the names in the genome versions GCA_000005845.2 and R64-2-1^67^, respectively, and gene names without match with these genome versions were excluded. The filtering steps consisted of selecting only the “confirmed” labeled interactions of *A. thaliana* and the “transcriptional directed” labeled interactions that matched Uniprot identifiers of *D. melanogaster* and humans. Additionally, GRNs of embryonic stem cells of mouse assessed by ChIP-ChIP and ChIP-Seq (Table 1) were downloaded to be used as test sets (further detailed).

After filtering, the genes and regulatory relationships were modeled as nodes and undirected links, respectively. Thus, we assessed the node degree distribution of each filtred species-specific GRN to check their reliability. Each GRN degree distribution was fitted using a power-law function (${P}_{deg}(k)\propto {k}^{-\gamma }$), and the coefficient of determination (*R*^2^) was calculated.

For machine learning purposes, genes and topological features are called instances and attributes, respectively. The topological GRN features (attributes) were calculated before the attribute selection, test set selection, modeling, and test set classification (further detailed). We used the Igraph package^68^ implemented in R^69^ to calculate the eccentricity, degree, eigenvectors, betweenness, closeness, page rank, strength, hub score, coreness, subgraph centrality, burt constraint, transitivity, and the average nearest neighbor degree (K_nn_) topological features of each gene (instances); this process was performed for each GRN independently. Afterward, values of each attribute were discretized into 6 bins (“A”, “B”, “C”, “D”, “E” or “F”) for each GRN (individually) using the standard deviation (σ) binning method^70^ as follows: $A\le \underline{{{x}_{k}}}-2{\sigma }_{k}$; $\underline{{{x}_{k}}}-2{\sigma }_{k}<B\le \underline{{{x}_{k}}}-1{\sigma }_{k}$; $\underline{{{x}_{k}}}-1{\sigma }_{k}<C\le \underline{{{x}_{k}}}$; $\underline{{{x}_{k}}}<D\le \underline{{{x}_{k}}}+1{\sigma }_{k}$; $\underline{{{x}_{k}}}+1{\sigma }_{k}<E\le \underline{{{x}_{k}}}+2{\sigma }_{k}$; and $F>\underline{{{x}_{k}}}+2{\sigma }_{k}$, where $\underline{{{x}_{k}}}$ is the mean and ${\sigma }_{k}$ is the standard deviation of the values of an attribute $k$. The *cut* function divides the entire value range into bins, and the range covered by each bin (e.g., the bin size) was uniform. Values assigned as “inf” during conversion were stated as “missing information” (“NaN”) to allow the learning.

Each instance (gene) was labeled as “regulators” or “targets” (the instance’s class) according to the databases information; this step is crucial for supervised learning. A total of 406 regulators in species-specific GRNs are repeated as targets, and the ones were maintained in the datasets since it is a common feature of GRNs^14^; furthermore, our initial assays showed no relevant impact removing these genes.

A total of 10% of regulators and the same number of targets from species-specific GRNs were randomly selected to compose test sets. The full GRN from mouse embryonic stem cells were also used as test sets. The test set instances were set up as “unlabeled” and were not used to generate the classification model (the training steps). Therefore, since the test sets have model-unseen instances, they were used to evaluate the predictive performance of consensus classification models and its generalization trends (further described). The rest of the data composed the training set.

The number of targets overcomes the number of regulators in the training set. Then, we performed an undersampling of both regulators and targets to create balanced datasets to avoid degeneration on training performances^71^. For this purpose, the target instances were randomized, followed by splits into several smaller sets proportional to the regulators. The regulators were further inserted into all those smaller sets creating 12 balanced training sets. Then, instances within each training set were randomized before training to avoid bias during the cross-validation step (Supplementary Datas S1, S2). Random training sets from the normal sets were obtained shuffling only the class.

### Attributes selection, supervised learning, and gene ontology analysis

The attributes selection and the machine learning steps were performed using Weka^72^ v3.8.5. For the model simplification to avoid overfitting, the most informative attributes were selected from a matrix with the whole species-specific sets (training plus test sets) by the BestFirst (greedy hillclimbing with a backtracking facility) and CfsSubsetEval (-D 1 -N 13) (select attributes that are highly correlated with the class but low intercorrelated) algorithms, which were also supported by the Ranker and InfoGainAttributeEval algorithms. After defining the main attributes (K_nn_, page rank, and degree), the ones were selected in each training and test sets before the learning and test set classification. The degree of the node *i*, *k(*_*i*_*)*, is its number of connections. The K_nn_ of a node *i* is related to each neighbor’s degree (*k(j)*): ${K}_{nni}=\frac{1}{k(i)}\sum_{j}k(j)$. The mathematical background of the page rank estimation is not trivial because the one is recursively defined: the page rank of a given node relies on the page rank of all neighbor nodes^73^.

The classification models were generated for each balanced training set considering only the top 3 relevant attributes mentioned using the J48 (20 objects per leaf) algorithm with tenfold cross-validation; therefore, we could assess the relationship among these attributes considering regulators and targets. Then, a single normal consensus classification model was obtained using the Vote (-S 10 -R AVG) algorithm (Supplementary Data S3); the same modeling procedures were performed for the random sets generating the random consensus model.

The normal consensus model was used to independently classify each test set (the species-specific and embryonic stem cells) to assess the predictive performances over model-unseen instances and the generalization classification trends. The same procedure was performed using the random consensus model to evaluate the reliability of the normal model: in this case, the classification using the random model must present a much lower performance than the one using the normal model. The data distribution of predictive performances was evaluated using the Shapiro–Wilk test, and some data were not normally distributed. Then, the Mann–Whitney test was applied to evaluate the significance of differences between normal and random model performances within each dataset.

Individual decision trees from the training using the normal sets were evaluated to identify the relationship among the three most relevant GRN features, and the rules to classify regulators and targets were depicted in a consensus tree. The genes were split according to the classification tree's rules to explore the biological processes related to the genes that lie in the consensus tree leaves (Fig. 2a). For instance, if a given gene has a K_nn_, page rank and degree equal “C”, the one is a target that lies in a leaf end-branched by the degree (Fig. 2a); hence, the gene ontology (GO) terms of this gene will be at the “Degree tar.” column in Fig. 2b. All GO terms available for these genes were retrieved from UNIPROT and summarized using the REVIGO (no specific organism selection, “some other quantity, where” and “higher is better”)^74^.

### Simulation of GRN evolution

In order to assess which network perturbations contribute to the most important topological parameter ranked in the decision trees (the K_nn_ attribute), simulations were performed based on the equation of K_nn_ (Ref.^30^ over one regulator (the yellow node in Fig. 2c). The simulation starts from a small hypothetical network with 10 nodes and 9 edges (Fig. 2c I); this network also has 2 nodes with degree = 5 to represent potential regulators or simulating the targets controlled by multiple regulators, or even the duplication of downstream regulators (Fig. 2c I–IV). Then, we simulated pervasive transcription (Fig. 2c II), target duplication for a given regulator (Fig. 2c II,III), regulator duplication (Fig. 2c IV), and the degree increases of regulator's neighbors (Fig. 2c III,IV). Altogether, we hypothesized that gene duplication would contribute to the K_nn_. Thus, based on the first network (Fig. 2c I), we raised only the degree of the regulator (representing a target gene duplication) and, independently, we raised only the targets’ degree (representing a regulator duplication) (Fig. 2d).

## Supplementary Information

Supplementary Information 1.

Supplementary Information 2.

Supplementary Information 3.

Supplementary Figures.

Supplementary Tables.
