# Supplementary material for: Three topological features of regulatory networks control life-essential and specialized subsystems
Source: Sci Rep. 2021 Dec 20;11:24209. doi: 10.1038/s41598-021-03625-w (PMC8688434; doi:10.1038/s41598-021-03625-w)
Supplement: Supplementary file 1 — Supplementary Information 1. [file 41598_2021_3625_MOESM1_ESM.zip › Supplementary Data S1/Caption Supplementary Data S1.docx]

**Supplementary Data S1:** Training sets used to generate the models presented as an arff file format and all decision trees.
