# Supplementary material for: Three topological features of regulatory networks control life-essential and specialized subsystems
Source: Sci Rep. 2021 Dec 20;11:24209. doi: 10.1038/s41598-021-03625-w (PMC8688434; doi:10.1038/s41598-021-03625-w)
Supplement: Supplementary file 2 — Supplementary Information 2. [file 41598_2021_3625_MOESM2_ESM.zip › Supplementary Data S2/Caption Supplementary Data S2.docx]

**Supplementary Data S2:** Training sets used to generate the random models presented as an arff file format.
