# Supplementary material for: Three topological features of regulatory networks control life-essential and specialized subsystems
Source: Sci Rep. 2021 Dec 20;11:24209. doi: 10.1038/s41598-021-03625-w (PMC8688434; doi:10.1038/s41598-021-03625-w)

Supplementary Figures

Supplementary Figure S1: Degree distribution of each GRN used in the training set. The right table shows the coefficient of determination.

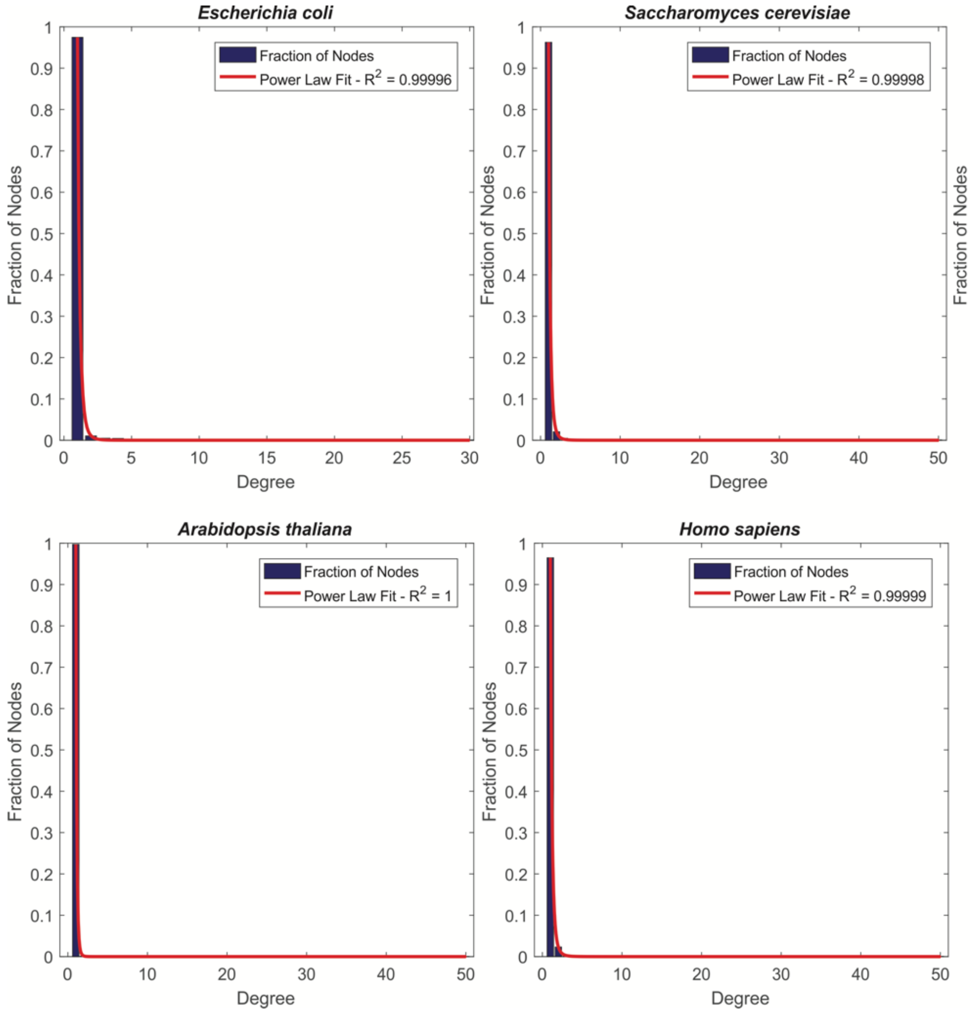

Species	R^2 from Power Law Fit
Escherichia coli	0.99996
Saccharomyces cerevisiae	0.99998
Drosophila melanogaster	0.99765
Arabidopsis thaliana	1.00000
Homo sapiens	0.99999

Supplementary Figure S2: Surface graphic and histograms of attributes K_{nn} *vs.* degree *vs.* page rank for the genes classified as targets and regulators for each species under evaluation. Targets have a more extensive range of K_{nn} values than regulators, such as reported in the first level of the consensus tree (**Figure 2a in the main text**). In the *Saccharomyces cerevisiae*, *Arabidopsis thaliana,* and *Escherichia coli* GRNs, the targets do not have nodes with both high K_{nn} and high page rank, such as the second level of the decision tree. Finally, in the *Drosophila melanogaster* and *Escherichia coli* GRNs, the range of degree values for targets is considerably lower than the regulators' values.

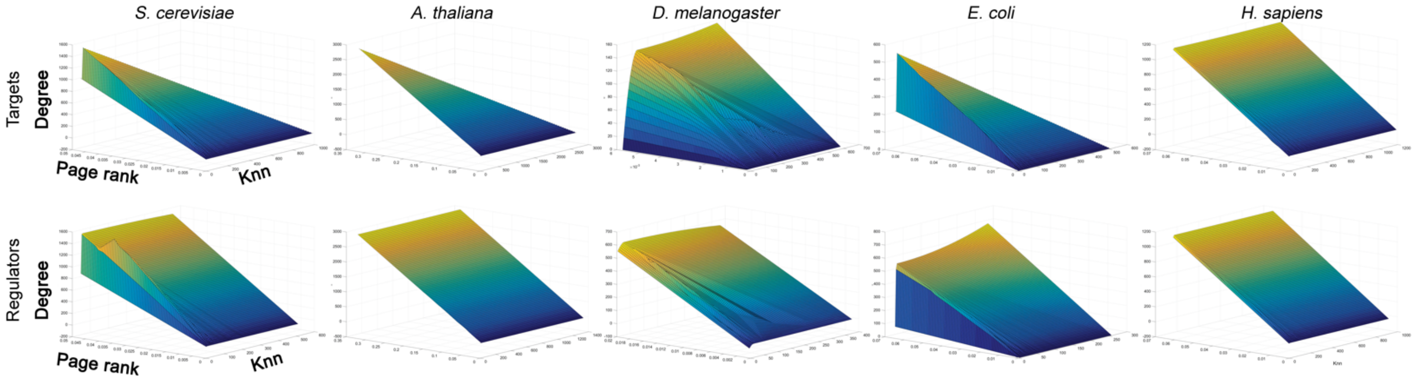

Saccharomyces cerevisiae

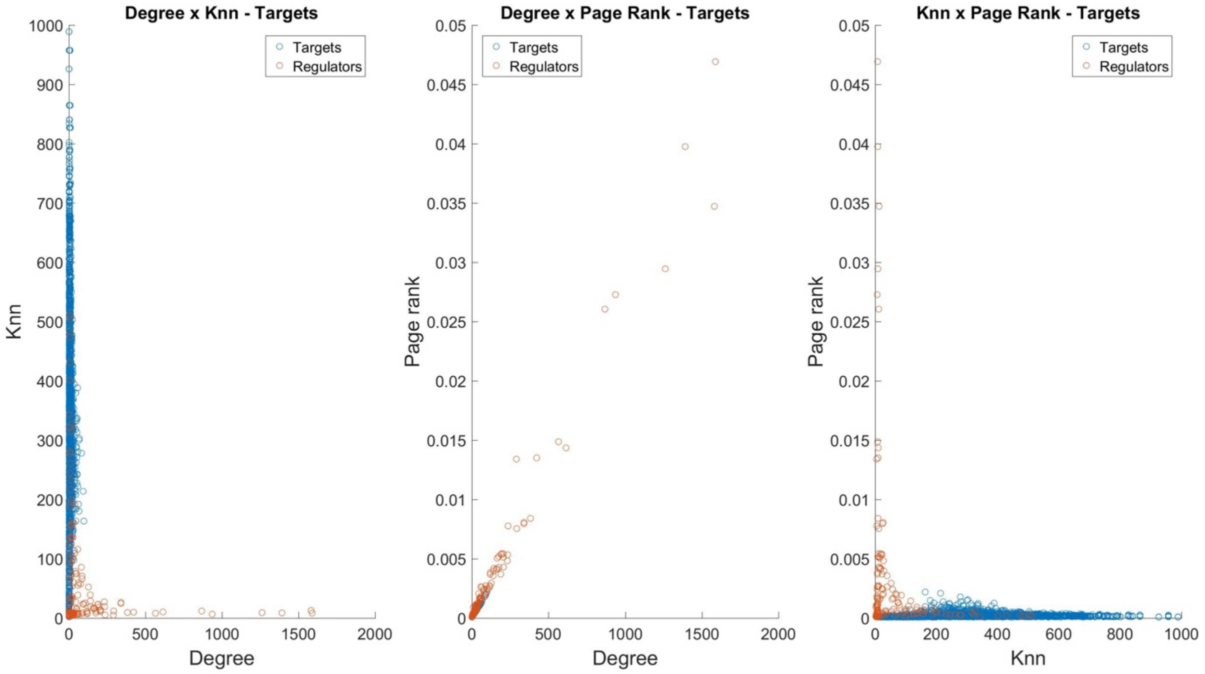

Arabidopsis thaliana

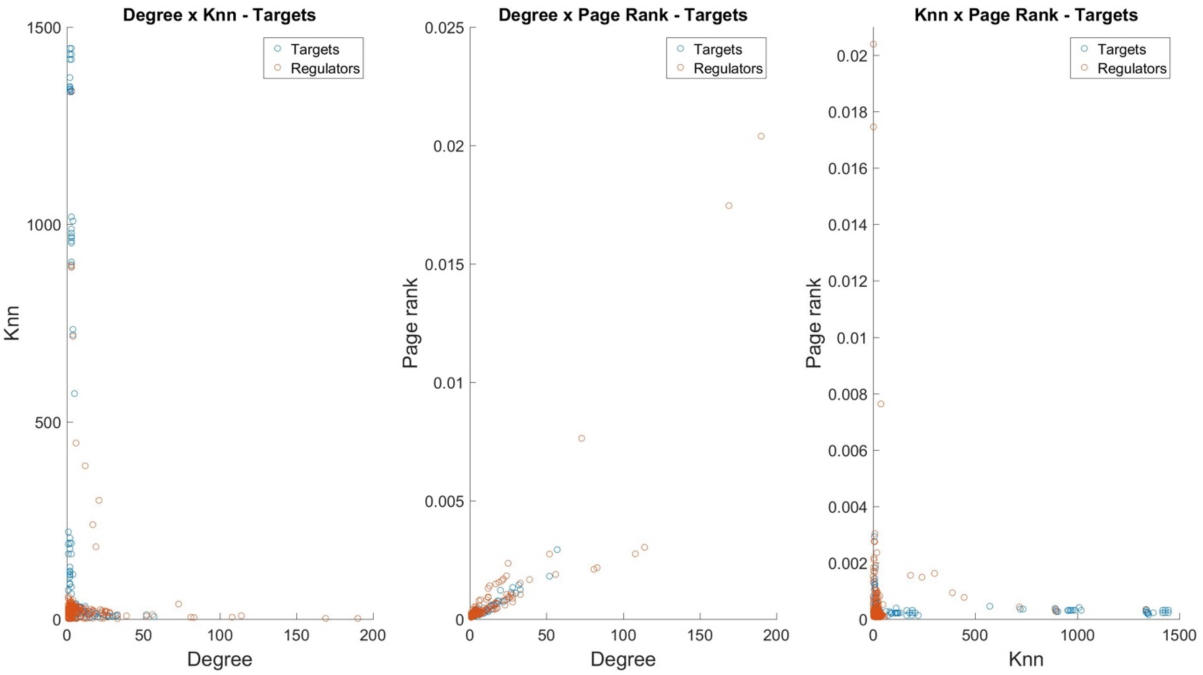

Drosophila melanogaster

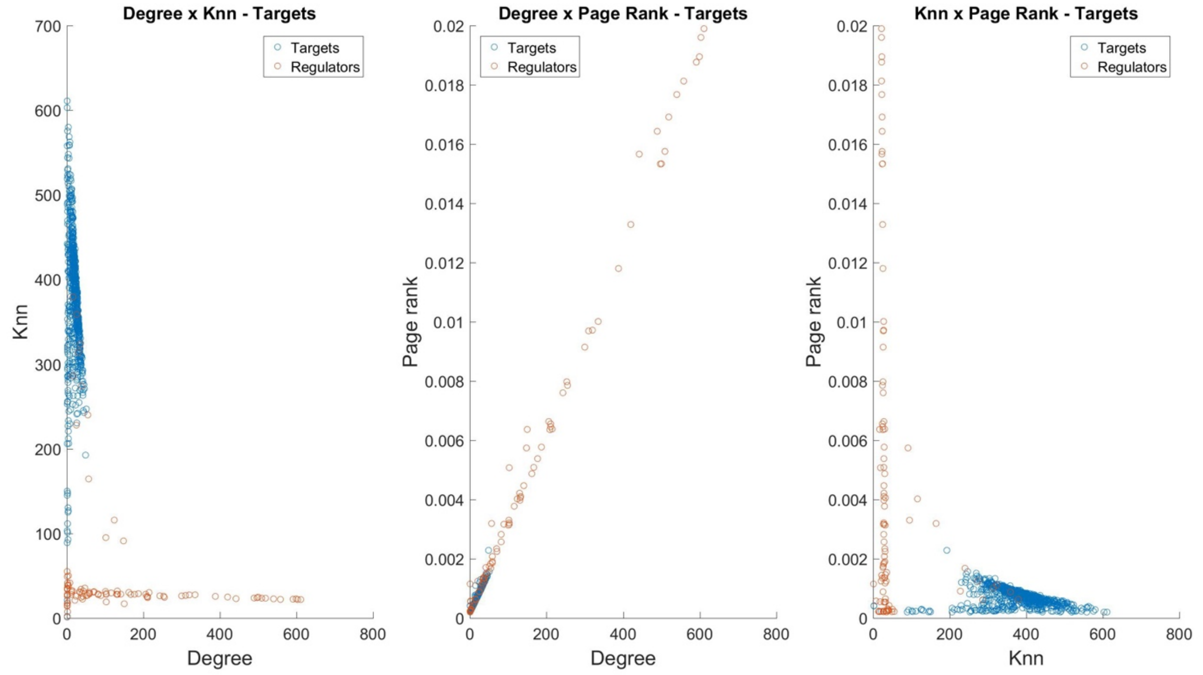

Escherichia coli

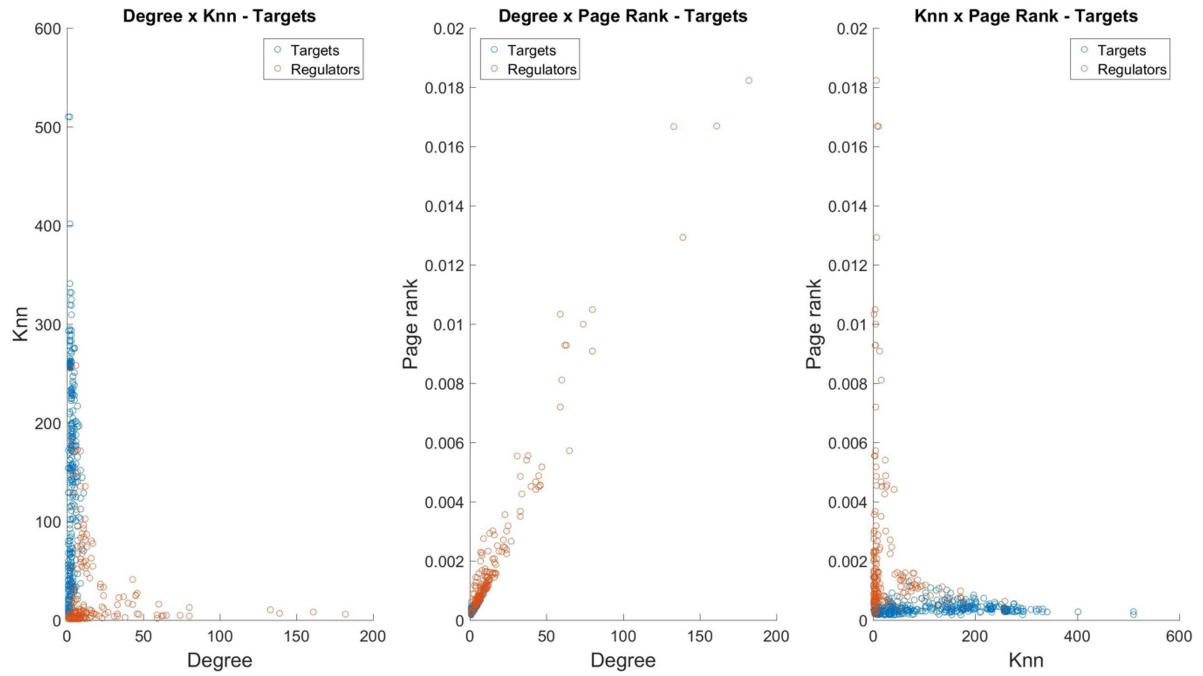

Homo sapiens

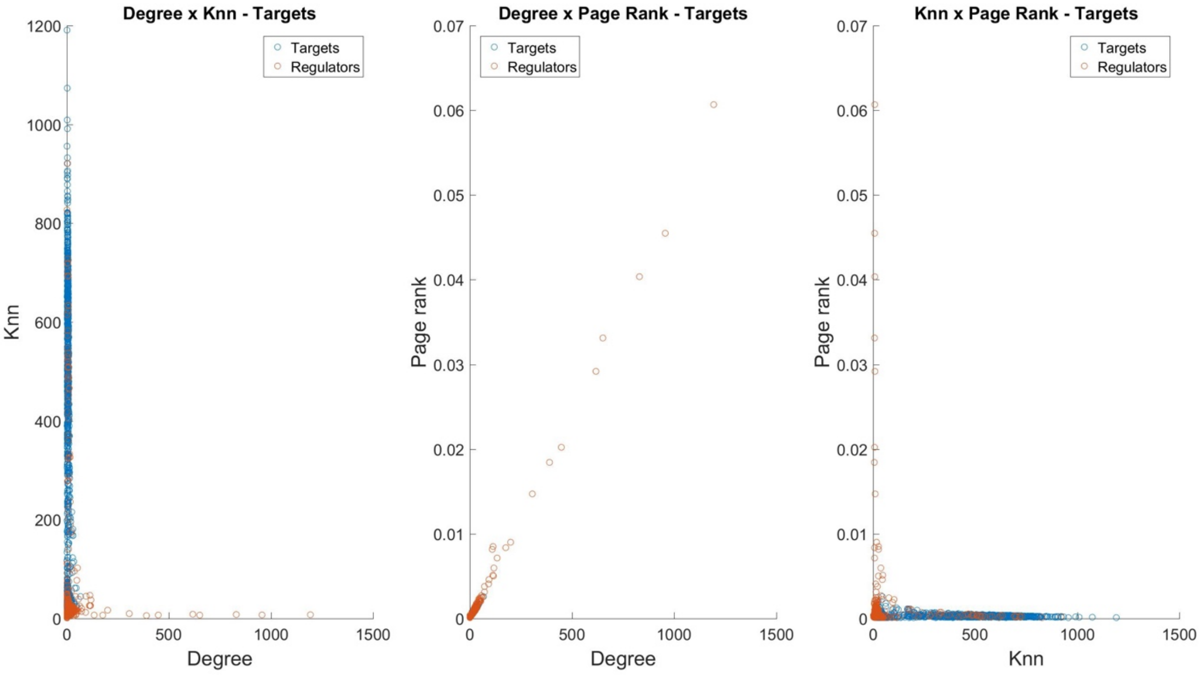

Supplementary Figure S3: All GOs sumarized by REVIGO.

K_{nn} reg.

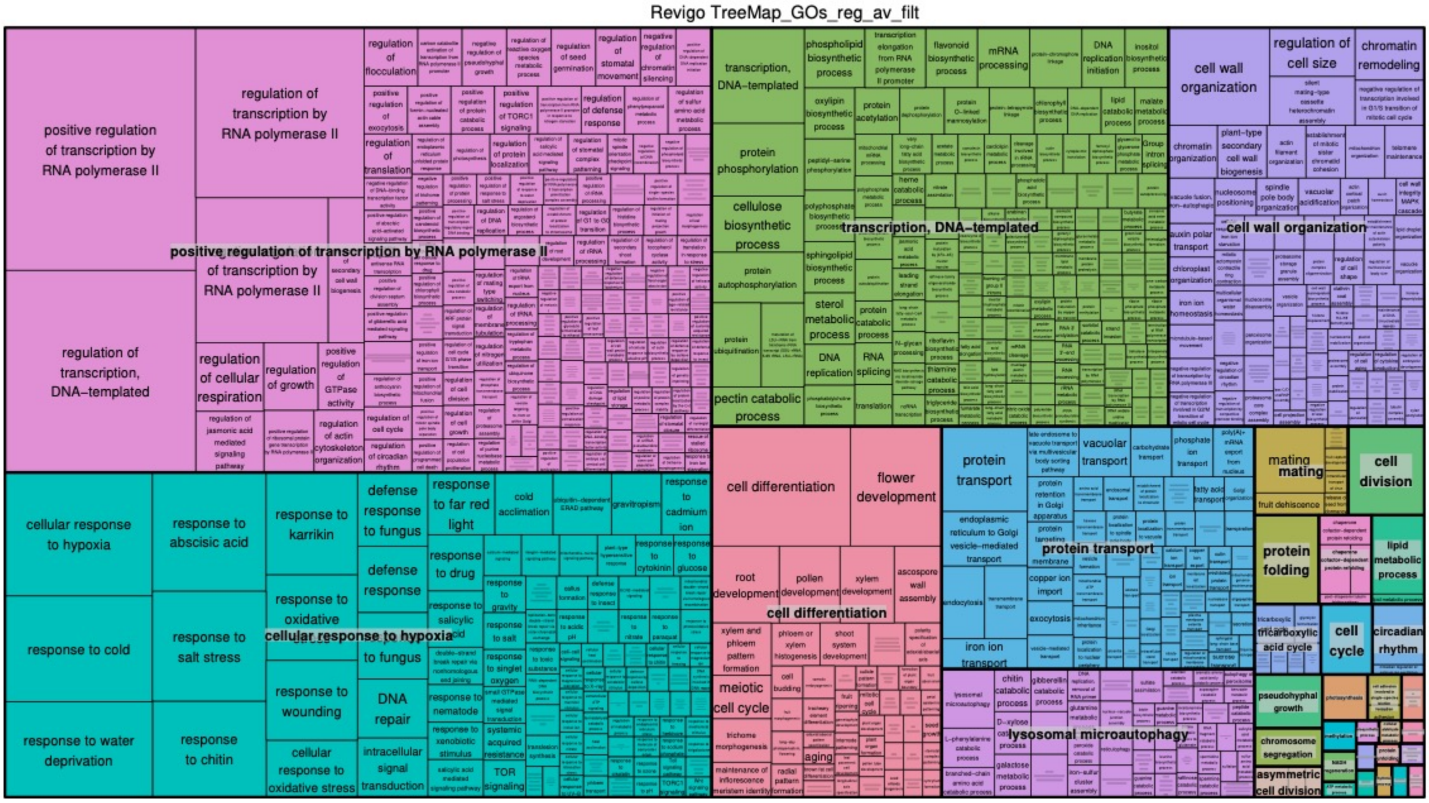

K_{nn} tar.

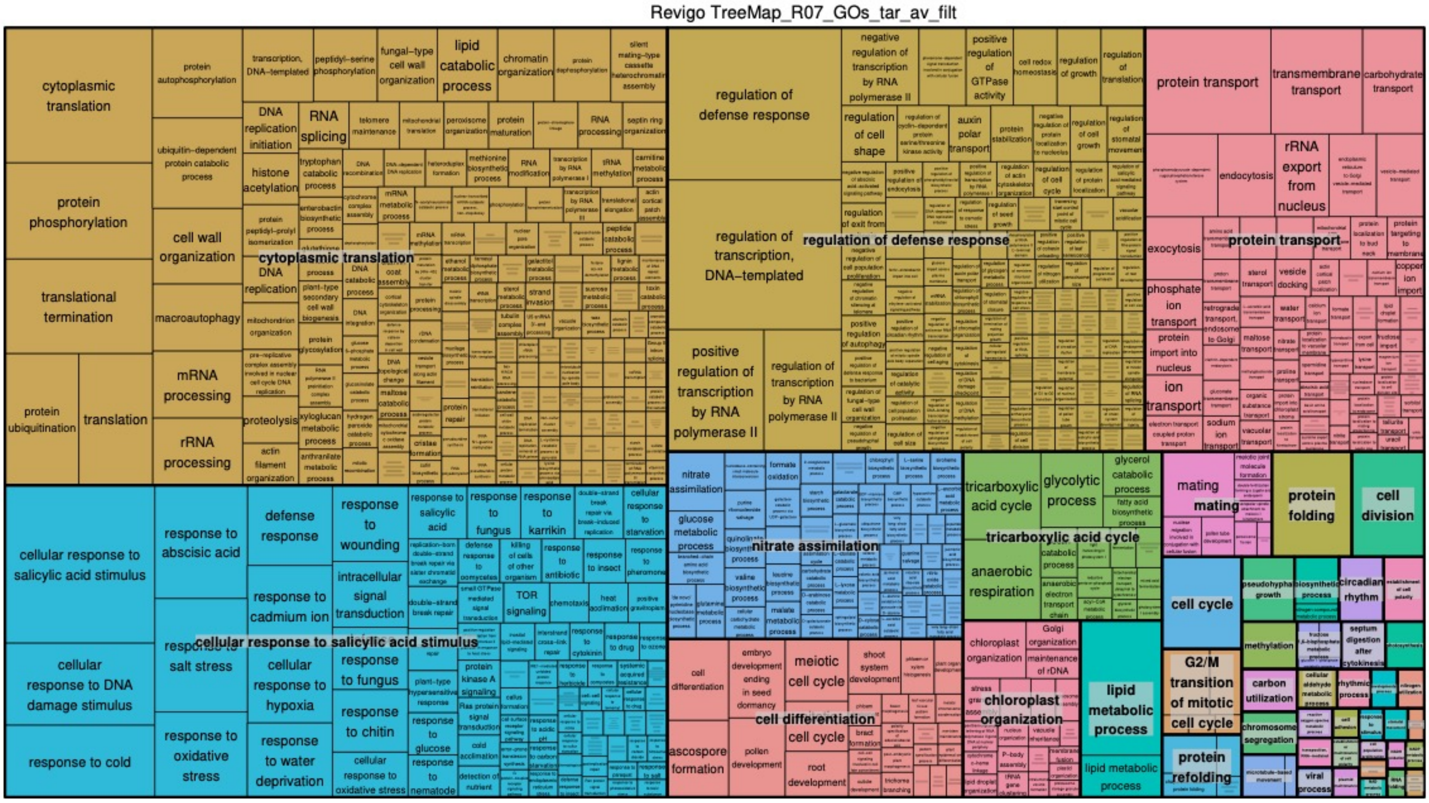

Page rank reg.

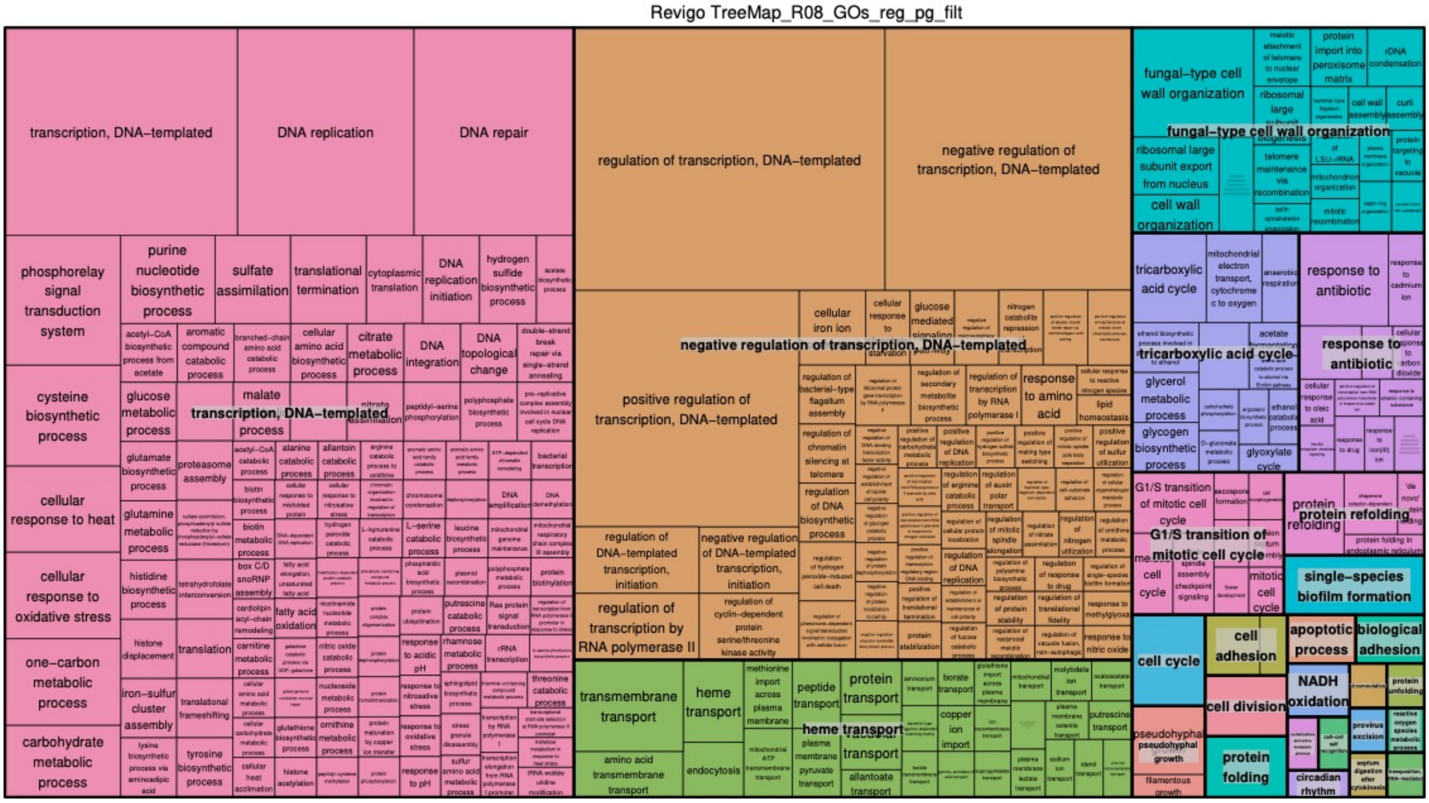

Degree reg.

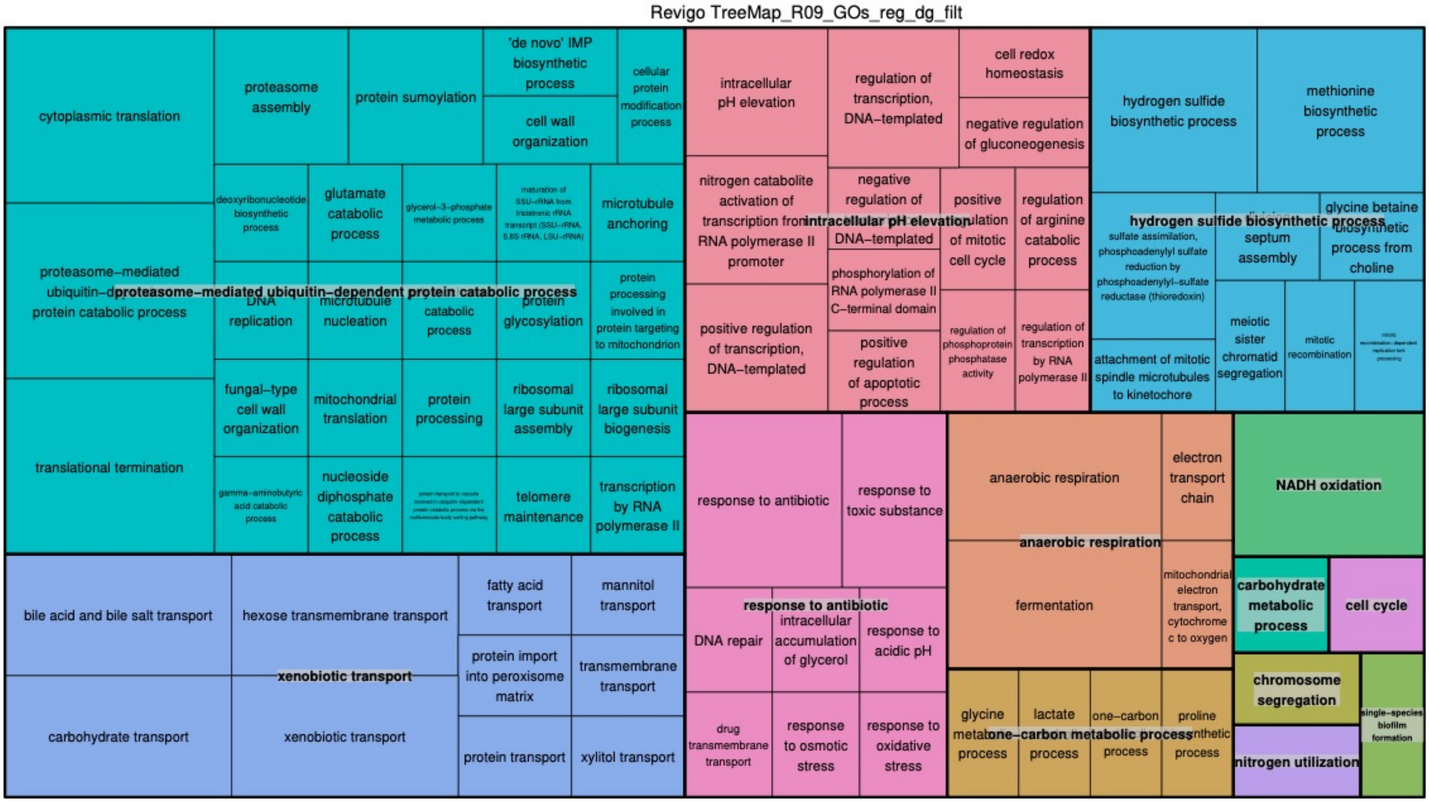

Degree tar.

Revigo TreeMap_R10_GOs_tar_dg_filt

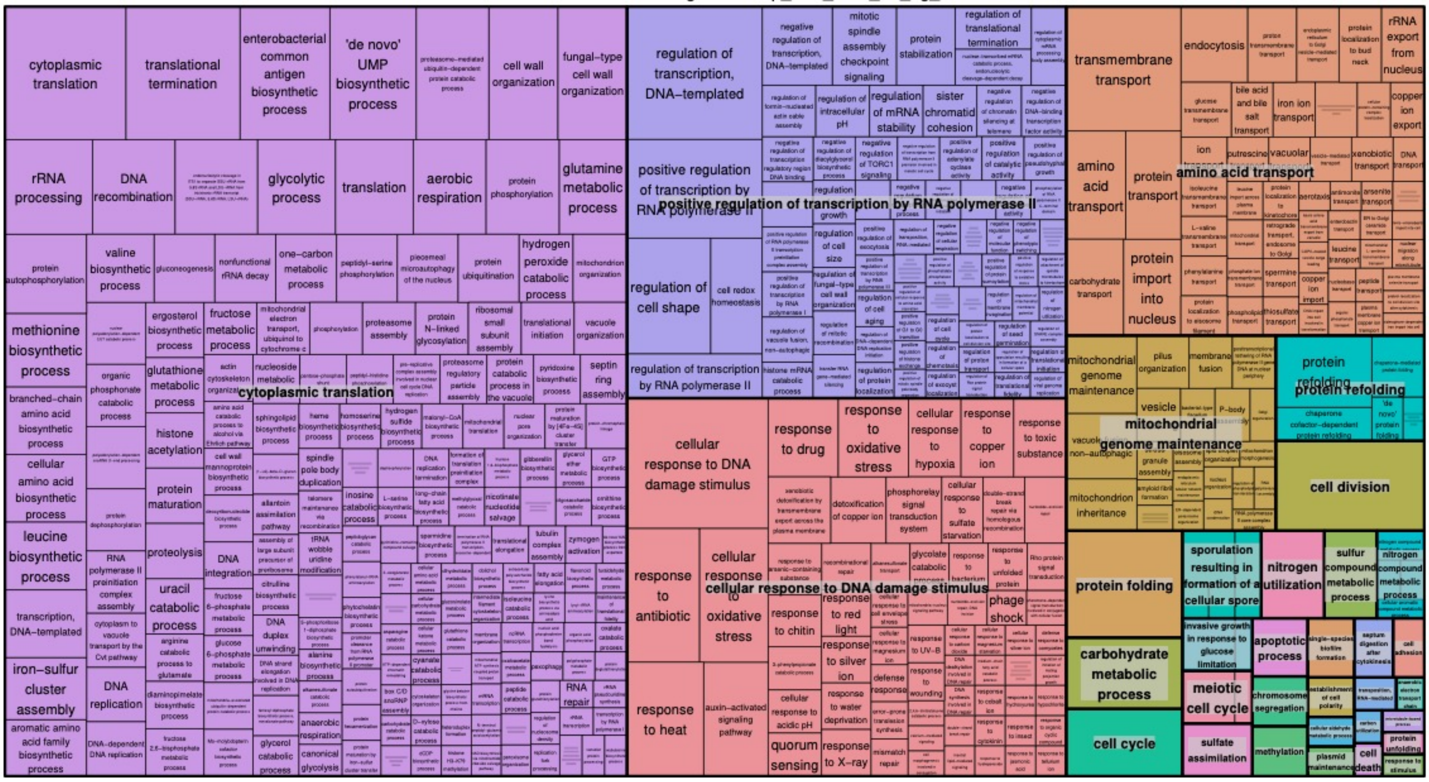

Supplement: Supplementary file 4 — Supplementary Figures. [file 41598_2021_3625_MOESM4_ESM.pdf]
